# The Impact of Cybervictimization on the Self-Management of Chronic Conditions: Lived Experiences

**DOI:** 10.2196/40227

**Published:** 2023-08-25

**Authors:** Zhraa A Alhaboby, Hala Evans, James Barnes, Emma Short

**Affiliations:** 1 The Open University Milton Keynes United Kingdom; 2 Coventry University Coventry United Kingdom; 3 Fatima College of Health Sciences Abu Dhabi United Arab Emirates; 4 London Metropolitan University London United Kingdom

**Keywords:** chronic diseases, biographical disruption, long-term conditions, self-management, cybervictimization, cyber abuse, cyberbullying, cyber harassment, disability discrimination, discrimination, awareness

## Abstract

**Background:**

Cybervictimization of people with long-term conditions is a disturbing phenomenon with a documented impact on health and well-being. These experiences are primarily examined using quantitative methods, focusing on children and young people. However, research centered on the cybervictimization of adults with chronic conditions is scarce, with limited qualitative input from the *victims* as experts in their own experiences.

**Objective:**

This study aims to understand the impact of cybervictimization on the self-management of long-term conditions among adults with chronic conditions and disabilities in the United Kingdom.

**Methods:**

This paper reports the findings from the qualitative phase of a phenomenologically informed mixed methods study. The biographical disruption concept was used to conceptualize the study. In-depth semistructured interviews were conducted with 13 participants with chronic conditions who experienced cybervictimization. A codebook was developed, and a zigzag approach to thematic analysis was used to define and refine themes. Ethical considerations and risk assessment were ongoing during the research process because of the sensitivity of the topic and cases of harassment.

**Results:**

Cybervictimization has direct and indirect impacts on the self-management of chronic conditions. This impact was verified across 6 overarching themes that emerged from this study. First, biomedical events included overall health deterioration because of existing conditions, new diagnoses, and subjective physical complaints. Second, the impact on mental health was perceived through psychological consequences and psychiatric disorders that developed after or during this traumatic experience. Third, the multilevel impact theme focused on disrupting the strategies for coping with health conditions and involved unplanned changes to victims’ health management priorities. Fourth, the impact of complexity reflected the perceived uniqueness in each case, intersectionality, struggle to obtain formal support, and subsequent health complications. Fifth, social network involvement comprised the effects of social isolation, victim blaming, and deception. Finally, the disability discrimination theme focused on prejudice, issues on inclusion, and hostility in society, with subsequent effects on well-being.

**Conclusions:**

People with long-term conditions experienced different forms of cybervictimization, all disruptive with various effects on health. Disability discrimination was a prominent finding to be further investigated. This paper reports the impact as themes to guide further research and practice, with the recognition that long-term conditions and impairments are not a homogeneous group. Despite the devastating consequences, there are positive points that strengthen potential interventions. Awareness-raising campaigns, training of support channels, and multidisciplinary research are recommended to tackle this issue and initiate change.

## Introduction

### Background

Chronic health conditions are becoming increasingly prevalent worldwide [1-3]. These are health conditions characterized by long durations, often for life, and the need for consistent self-management. Such health conditions overlap with disabilities; however, not all chronic conditions are disabling, and not every impairment is a chronic condition. To clarify this overlap, it is estimated that 25% of people with chronic illnesses have disabilities and that 80% to 90% of individuals with disabilities have long-term conditions [4]. In the United Kingdom, individuals with long-term conditions comprise approximately 30% of the population, 64% of outpatient clinic appointments, and 70% of hospital stays [5]. Meanwhile, 22% of the population had a disability from 2020 to 2021 [6]. Disability is defined by the Equality Act 2010 as a “physical or mental impairment and the impairment has a substantial and long-term adverse effect on [an individual’s] ability to carry out normal day-to-day activities” [7]. In this study, long-term conditions and disabilities were included, and the definition used was a long-term health condition or impairment with a duration of no less than 3 months that is likely to last more than a year. The definition of a long-term condition in this study also involved self-management.

Self-managing chronic conditions is an evidence-based approach to preventing complications and achieving better health outcomes. It involves patients in their own health [8]. Individuals living with chronic conditions believe that such management plans help bring order to their lives and enable them to cope with their long-term conditions [9]. Managing each chronic condition depends on its nature, so each plan has a different goal. For example, medication adherence and exacerbation prevention are the primary targets when managing asthma. Meanwhile, in the case of diabetes, the focus is on regulating blood glucose levels and maintaining lifestyle modifications [10]. Therefore, self-management requires commitment and is often physically, emotionally, intellectually, and socially demanding [11].

Reaching optimum health outcomes when managing chronic conditions is barely achievable because of the influence of other complex factors [10]. A systematic review examined self-management interventions in individuals aged 7 to 25 years in 86 studies [12]. It was concluded that most studies focused on the medical input of self-management, whereas the psychosocial aspect was overlooked [12]. This continues to be an issue, as demonstrated in a more recent systematic review that included a qualitative synthesis of 14 articles and examined health encounters with patients with long-term conditions [13]. It was found that medical information was dominant in these clinical encounters, with little attention paid to the psychosocial aspects of health management. Accordingly, the self-management of chronic conditions can be disturbed when psychological resources are depleted [8], and one factor potentially disturbing these resources is cybervictimization.

The victimization of people with long-term conditions is an existing phenomenon in an offline context [14,15]. Distress and considerable subsequent impacts on mental well-being are commonly associated with such experiences. However, web-based communication has facilitated such victimization and introduced this phenomenon’s potentially more complex cyber variation [16,17]. Furthermore, cybervictimization has consistently been linked to poor mental and psychological outcomes among young people [18]. This makes cybervictimization a relatively new risk that directly depletes psychological resources and influences self-management when the target is a person coping with a long-term condition.

A systematic review examined the documented impact of cybervictimization on people with long-term conditions [19]; the narrative synthesis of reported results covered 10 studies on 3070 individuals with chronic conditions or disabilities. The sample sizes ranged from 42 to 823 participants, and the age range was 6 to 71 years. However, 9 of the studies had participants aged <20 years. The reported prevalence range of cybervictimization was 2% to 41.7% [19].

The terminology used to define web-based experiences of people with long-term conditions was inconsistent. These experiences included cyber harassment, cyberbullying, cyberstalking, or cyber hate incidents. The details of each case, the discipline, and the country where the study took place influenced the terminology [19]. Cyber harassment was characterized by intimidation and threats from a person or group to another individual or group, whereas cyberstalking was more fixated and persistent. Cyberbullying was mentioned when there was a perceived power imbalance between the perpetrator and the target, meaning situations in which the targeted person could not defend themselves or the perpetrator had more power, such as in the workplace or school. Disability hate was documented when the targeted person was disabled, and the bullying was perceived to be motivated by hate. *Cybervictimization* was commonly used as an umbrella term to describe intimidation or abuse experienced through web-based communication [20,21]. A recent review [22] described the constant challenges in finding consistent definitions and, in turn, providing a specific estimate of the scope of cybervictimization. Owing to the inconsistencies in definitions, the term *cybervictimization* was used to cover negative web-based communication, as described in the *Methods* section.

Despite the differences in definitions, cybervictimization’s impact was consistent. The reported effects, specifically concerning people with long-term conditions or disabilities, included depression [20,23-28], anxiety [23,25,27-29], suicide or self-harm [25,27,28], low self-esteem [23,24], behavioral issues [29], and substance abuse [20].

In the United States, a cross-sectional study examined the impact of bullying and cyberbullying on adolescents with asthma in a sample of 6212 high school adolescents [26]. Cyberbullying was reported by 243 (8.3%) boys and 529 (16.6%) girls. This was associated with depressive symptoms in 550 (18.7%) boys and 1074 (32.9%) girls. There was a significant relationship between having asthma and experiencing cyberbullying (*r*=−0.056; *P*<.01) and depressive symptoms (β=1.43; *P*<.01) in adolescents.

One of the noticeable gaps in the cybervictimization literature was the lack of qualitative research. All the studies in the aforementioned systematic review [19] primarily adopted quantitative methodologies, which remains an issue [18]. Furthermore, the criteria for identifying people with chronic conditions or disabilities potentially directed the previous studies’ results and introduced disciplinary variations. These included psychology, psychiatry, health sciences, public health, criminology, and social work. However, only 2 studies were from a public health perspective [21,27]; these were conducted in Sweden and reported more detailed physical and mental health–related variables. A cross-sectional study [27] was conducted with 413 children aged 13 to 15 years with chronic conditions or disabilities. Cybervictimization was reported in 33.5% of the bullied children with disabilities or chronic diseases in the previous 2 months. The researchers used a comprehensive list of health indicators, which included poor general health, physical health, mental health indicators, and self-harming behaviors.

Another cross-sectional study [21] was conducted with children aged 12, 15, and 17 years. The sample included 762 children with disabilities. The scope of cyber harassment was 14% among boys and 20% among girls. The reported impact addressed general symptoms grouped under *subjective health complaints* using a validated instrument—the Health Behavior in School-aged Children Symptom Checklist. The participants’ health status was determined through responses to questions on headache, feeling low, irritability, nervousness, sleep disturbances, and dizziness.

The quantitative phase of this research examined a sample of 152 participants with long-term conditions in the United Kingdom [30]. In this sample, 45% (69/152) of the participants experienced cybervictimization, and 61.1% perceived a negative impact on the self-management of their health. Most survivors (53/69, 77%) were disabled, and the relationship between cybervictimization and disability was statistically significant (*P*=.03). In addition, the recognized impact on health was statistically significant for a long duration of harassment (*P*=.03). This relationship required further exploration and gathering of firsthand experiences from the survivors to understand what led to these figures.

At the time of conducting and reporting this research, there was no similar study carried out in the United Kingdom despite the documented cases of cyber abuse [16] and the efforts to examine the practices [31] and the law [32] to efficiently address the role of technology in facilitating this issue. In addition, a recent review of evidence carried out by a research team on behalf of the UK Council for Internet Safety acknowledged the lack of research examining the harms of web-based hatred toward adults with disabilities in the United Kingdom [33,34]. This study inspected cybervictimization experiences among people with long-term conditions in the United Kingdom. This study’s findings will provide insight into the experiences from the *victims’* perspectives to enhance formal support and raise awareness of this phenomenon.

### Theoretical Background

This section discusses the theoretical background underpinning this study. It links the biographical disruption concept; the self-management of chronic conditions; and cybervictimization as a traumatic, potentially disruptive event.

#### Chronic Conditions as “Biographical Disruptions”

Once a person is diagnosed with a chronic condition, every aspect of their life is susceptible to change. Bury [35] proposed conceptualizing chronic conditions as *disruptive events* in an individual’s life. This proposal was a development on what Giddens [36] described as a *critical situation* in their discussion of major events that disrupt society, such as wars. Chronic conditions can be seen as a form of critical situation at an individual level; hence, they are described as a *biographical disruption* [35]. Such disruption involves three main characteristics: (1) disruption to thinking of *taken for granted* as, once the individual is diagnosed with a chronic disease, health and lifestyle cannot be taken for granted as compared with the previous state; (2) disruption to *explanatory systems*, which results in a rethinking of the self in which illness may become part of the person’s biography; and (3) the response to this disruption by moving *social and psychological resources* [35]. The concept of biographical disruption was challenged by Williams [36] in a critical reflection; biographical disruption was criticized for focusing on newly diagnosed conditions and the uncertainty surrounding them in an individual’s life. Consequently, this could mean a lack of the accounts of very young patients who were born with these conditions or were diagnosed with them so early that their condition became the norm [37]. That is why further exploration of this concept was recommended to include both ends of the life course. However, Larsson and Grassman [38] disagreed with this notion based on the findings from 2 large qualitative studies from a life course perspective. One study was prospective and involved interviews with patients with chronic illnesses and visual impairments secondary to diabetes or congenital defects over 30 years from 1981 to 2011 in Sweden. The other was a retrospective study that included older patients who lived with chronic conditions for a long time, such as multiple sclerosis, poliomyelitis, spinal injuries, and gastrointestinal conditions, and was supportive of the concept of biographical disruption, further adding that the onset of chronic illness was not the only disruptive event in these patients’ lives. The study also implied that developing complications and physical impairments during disease progression represent continuous disruptive events even if they were expected when the natural course of the disease was known [38].

One of the earliest proposed conceptualizations of health conditions considered the biomedical event as the disease, whereas the subjective personal experience is the illness and the social representation of the disease is the sickness [39]. Lonardi [40] combined this conceptualization with the concept of biographical disruption and applied them to chronic primary headaches, including migraine and cluster headaches [40]. The invisibility of chronic headaches resulted in *identity negotiation* to avoid stigma between the subjective experience (the illness) and disclosing the condition to others (the sickness).

One of the consequences of conceptualizing chronic illness as a disruptive event is the mobilization of psychosocial resources [35]. Social support is one of the primary recognized aspects of psychosocial resources [41]. It is a form of informal support that could reduce the effects of stress and prevent health deterioration. The model of social support was adopted to explain the cybervictimization of young individuals with long-term conditions. This was achieved through the main effect model, which considers that support is always helpful, and the stress-buffering model, which considers that support depends on the level of incident or stress caused [21]. It was found that social support is a buffer against the impact of cybervictimization on young people. In turn, barriers to social support seem to exacerbate the psychological impact of cybervictimization [42].

#### Biographical Disruption and the Self-Management of Chronic Conditions

Self-management is a dynamic process that emphasizes enabling individuals collectively with their family, community, and health care professionals to manage the chronic condition in terms of symptoms, treatment, lifestyle changes, and psychosocial support [43]. Hence, it is essential to differentiate it from self-care, which implies more generic and physical care. Researchers argue that self-management is rooted in the biographical disruption concept [44], specifically how biographical disruption influences the meaning of living with the illness and the meaning of the bodily consequences of having a long-term condition [35]. The self-management of chronic conditions shares with the biographical disruption model the concept of mobilizing psychological resources [8], which indicates that it is a stage in the disruption process and could be influenced by repeated disruptions, raising the question of whether another stressful event, such as cybervictimization, is experienced as a separate disruption or a continuous disruptive event in the chronic disease progression.

#### Cybervictimization and Biographical Disruption

Biographical disruption is based on the fact that the diagnosis of a chronic condition is a major event in life, and it was found that individuals who experienced cybervictimization perceived it as a major event that changed them forever [16,30,45,46]. Furthermore, it was found that the diagnosis of a chronic disease is sometimes associated with *posttraumatic stress disorder* (PTSD) [47]. This reflects the disruptive nature of living with long-term conditions. This also supports the argument of whether the concept of biographical disruption applies to cybervictimization, which is also considerably associated with PTSD [19,30,45,46,48].

Cybervictimization affects psychological well-being and disrupts social relationships, which are part of the psychological resources [42]. This is a core issue in chronic disease self-management [8] and one of the main building blocks covered in the biographical disruption concept [35]. Hence, mobilizing psychological resources is a common factor in cybervictimization, the self-management of chronic conditions, and coping with disruptive events in life. Moreover, if the diagnosis of chronic conditions changes the status of *taken for granted* related to health issues, the person has to live with the new diagnosis and the potential complications of the chronic condition, unlike before [35]. This might apply to cybervictimization as survivors showed “trust” in web-based relationships and took them for granted [49], whereas after the experience of cybervictimization, they become extremely cautious, isolated, and afraid [16,45,46].

**Figure 1:**
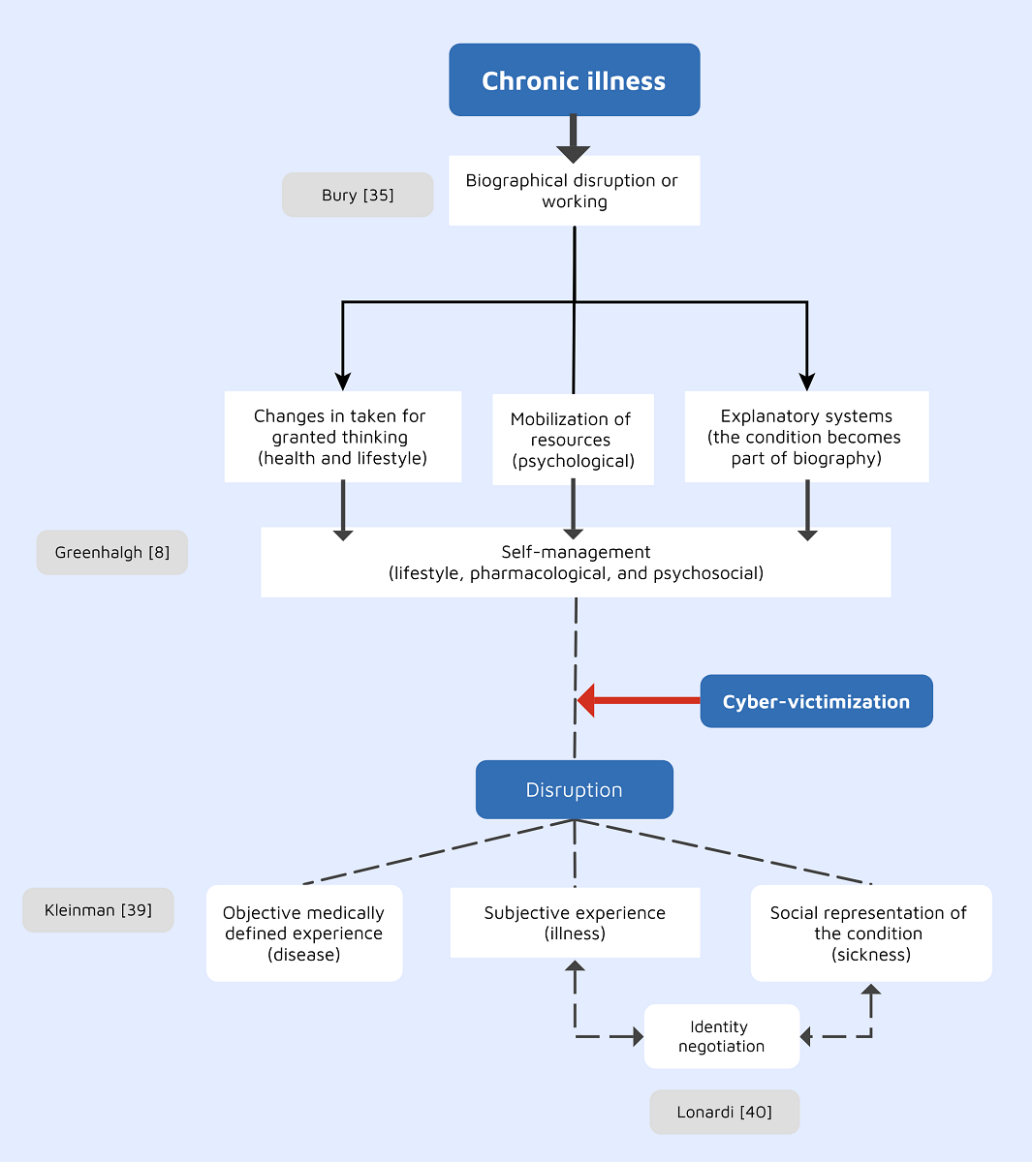
The theoretical framework underpinning this research [8,35,39,40].

Consequently, it could be assumed that cybervictimization “behaves” like a chronic illness in disrupting people’s lives. Thus, research is needed to explore this issue and examine whether cybervictimization acts as a disruptive event by itself or interacts with the preexisting chronic condition to result in a series of complications and how this could be addressed by professionals. On the basis of the aforementioned discussion, Figure 1 [8,35,39,40] was developed to illustrate the conceptual framework underpinning this study.

The framework proposes that living with a long-term condition is a disruptive event. It changes the thinking of self as well as the *thinking for granted.* It also requires mobilizing psychological resources. Once this happens, life will change, but the self-management that is rooted in this disruption starts to enable coping with the situation by mobilizing psychosocial resources. However, when being a target of cybervictimization, a second disruption could occur. This time, the disruption occurs on top of an existing condition and could have a different impact on the subjective, objective, or social experiences [39]. This impact could also vary based on identity negotiation by the survivor, who is influenced by psychosocial factors [40].

## Methods

In this paper, we report the findings of the qualitative phase of a phenomenologically informed mixed methods study to examine the impact of cybervictimization on the self-management of adults with long-term conditions [50,51]. A sequential mixed methods design was used in which the quantitative phase was followed by purposeful sampling in the qualitative phase.

### Ethics Approval

Ethics approval was sought from the Research Ethics Committee based at the University of Bedfordshire (IHRREC C557). The risk assessment was an ongoing process carried out by a multidisciplinary team owing to the sensitivity of the topic. After the interviews, the participants were debriefed, and the researchers offered a practical self-help book and signposted the participants to relevant support channels to provide both informational support and referrals if needed.

### Sampling of Participants

In a sequential mixed methods design, the sampling in the quantitative phase was followed by purposeful sampling in the qualitative phase, which is a well-documented approach [52-54]. Purposeful sampling is a type of nonprobability sampling that helps identify individuals with rich information that inform the knowledge about the research problem of interest [55]. Purposeful sampling can be based on “criterion I,” which means including participants who meet the predominant criteria of interest, or on “criterion E,” which means including participants who are outside the predominant criterion of interest. Other strategies include taking a homogeneous sample or taking the extremes in maximum variation sampling [55]. The “criterion I” approach was adopted in this study to include adults who were living with long-term conditions or impairments and had experienced cybervictimization.

### Inclusion Criteria and Definitions

The inclusion criteria were individuals aged ≥18 years, of any gender and ethnic background, with the capacity to consent according to the Mental Capacity Act 2005, living with long-term conditions, residing in the United Kingdom, and currently experiencing or having experienced cybervictimization.

To define cybervictimization in this research, we considered the inconsistency in defining cybervictimization cases in the literature. Hence, we asked the participants if they had experienced unwanted communication more than once via electronic means, such as email, chat room, web-based forum, social network, mobile phone message, or other web-based means, that was used to harass, insult, embarrass, or spread lies about them [19]. A chronic condition was defined as a long-term health condition with a duration of no less than 3 months and likely to last for >12 months. This duration is informed by medical criteria and the definition in the Equality Act [7]. At the time of data collection, the participants were experiencing or had experienced cybervictimization while living with a long-term condition in the United Kingdom.

### Semistructured Interviews

Qualitative interviewing is a flexible method for the in-depth exploration of people’s experiences and prioritizing the participants’ voices [56,57]. It helps address the lack of one-to-one interviews in cybervictimization research. An interview guide was developed to guide the participants with prompts throughout the interviews. The interview schedule started with taking the participants’ written consent and explaining their right to skip questions or withdraw. Participants were encouraged to talk first about their health conditions and self-management [8,58], how they felt when they were diagnosed, or how it felt to live with the condition or impairment [35,37,38]. This was followed by talking about the experience of cybervictimization and its impact. The motives of harassers were explored from the participants’ perspectives to investigate any perceived intentional targeting of people with chronic conditions or disabilities, as documented in the literature [59-61]. The prompts also included the impact of cybervictimization on health and self-management to ensure that the data were relevant to the research aim. Coping and support provided to survivors were also explored [62] to identify areas to be improved regarding support. At the end of the interview, participants were given the opportunity to talk about any further information they felt relevant to the study.

### Piloting the Interview Guide

The interview guide was developed following a discussion with experts in the field of cyber abuse and public health. The interview guide was piloted in 2 stages. Stage 1 was through obtaining feedback from a gatekeeper, who represented an online support group and was also living with a chronic condition and a survivor of cyber harassment. The gatekeeper suggested that the emotional impact should be further explored during the interview; this note was addressed in the questions. The second step included discussing the interview guide with 2 health researchers. The input from piloting was useful in ascertaining the participants’ understanding of the questions and the relevance of their input to the study. The discussion resulted in changes to the introduction to ensure that the participant understood the right to withdraw by asking specifically about this after the researcher’s introduction. The terminology was tailored to the participant’s preferred term in each case to describe the chronic condition or impairment. The prompts were rearranged chronologically, and an additional prompt—“What did you do next?”—was added.

### Recruiting for the Interviews

The participants were recruited for the interviews via the web-based survey in the quantitative phase [30,51]. In the last section of the questionnaire, participants who agreed to be interviewed had to tick a box and provide their email addresses or send an email to the researchers. The researcher responded by sending the participant information sheet and arranging a face-to-face or web-based interview based on the participants’ preferences.

### Interviewing Process and Transcription

Owing to the sensitivity of the topic, the researchers approached each interview with caution, and flexibility was required to address the participants’ health and emotional needs. The interviews were audiotaped. Some participants preferred a written conversation because of their health needs, such as memory issues (1/13, 8%) or being emotionally distressed (2/13, 15%), or because of practical issues (3/13, 23%). Audiotaping during qualitative data collection is a common practice to assist the researchers’ memory and note-taking ability [63,64]. However, it is acknowledged that it was not always possible, especially when considering the sensitivity of the topic. In the real-time interviews, the participants were asked whether they agreed to audio recording and note taking. All of them agreed to be audiotaped and provided verbal and written consent.

Participants were reminded of their right to withdraw and to share with the researcher if any question made them feel uncomfortable. Throughout the interview process, the researcher offered breaks, refreshments, and the option to skip questions. Following the interview, the interviewer offered contacts for support channels and a practical self-help book. There was flexibility in following the interview guide, but most participants talked freely, mostly rushing to talk more about their victimization experience and struggle for help.

Immediately after the interview, the interviewer wrote field notes to remember the most important aspects of the interview. This is a common practice in qualitative research to facilitate initial coding and ensure an understanding of the participant’s meaning [65]. The real-time interviews lasted between 30 and 90 minutes. Interviews were transcribed verbatim during the same week of the interview so that the participants could be approached if any questions arose during transcription. This approach also helped familiarize the researchers with the data and prepare them for analysis. The transcripts were anonymized, the lines were numbered, and the symbol *(...)* was used to indicate the tenths of seconds paused by the interviewees. The format of the transcribed interviews was Calibri, size 11, with single line spacing and 1 paragraph break between questions. A code was given to each participant in addition to the time and the keywords on the characteristics of the experience.

### Coding

The first step in coding was reading and rereading the transcripts several times before formal coding [66]. This was accompanied by keeping memos in the form of notes to self to highlight important notes, impressions, and problems that could be used later in the analysis and reporting [67]. The coding process was inductive because of the relatively scarce literature in the area and to allow for the identification of micro- and macroissues from the participants’ perspective.

Open coding was used by examining the data systematically. The researchers looked at the data and started coding line by line, interview by interview. A codebook was developed in response to the research question, which is an essential step in qualitative research to enhance credibility and reduce bias [63]. The codebook in this study was developed based on the guidelines provided by MacQueen [68]. The codebook consisted of code definitions, each one comprising the following: code label, brief description, where to use it, where not to use it, and an example from data extracts. The code label is a short prompt to distinguish codes from each other. The short definition is a descriptive phrase that captures the main components of the theme, whereas the full definition is a descriptive paragraph highlighting the key features of the code, such as conceptual or cultural dimensions. “When to use” highlighted the textual instances in which the code can be applied to the data, including double coding, whereas “when not to use” clarified the instances in which the code might not be applicable or overlapped with other codes [68]. In addition to these components, the researchers added a “relevance to the research question” label to ensure that the coding process fit within the research aim and avoids being distracted by the richness of the qualitative data [63].

Codes were manually applied to the data chunks by assigning a specific color. This stage of coding was inclusive; one data extract could be coded using more than one code. The coded data chunks varied between words, phrases, sentences, or paragraphs depending on the ideas shared by the participants and their relevance to the research question. After coding the data, codes were manually allocated to different papers under their specific colors. The codes were further examined in terms of recurrence and were refined into categories.

### Thematic Analysis

The 3 general aims of the analysis were to examine commonalities, differences, and relationships [69]. Thematic analysis was used because of its theoretical flexibility in this underexplored area. The thematic analysis guidance was followed in 6 steps [66]. The first was familiarization with the data through transcription, reading, and rereading. The second was the initial coding of all data systematically. The third was searching for themes by examining codes. The fourth was thematic mapping by reviewing and refining the themes. The fifth was defining and naming themes and subthemes. The sixth was writing up findings with sufficient evidence using narrative and data extracts. This process of data collection, coding, and analysis was implemented in a zigzag approach [67], which helped define and refine themes during data collection until the point of saturation of themes.

## Results

### Overview

A total of 13 in-depth interviews were conducted with individuals living with long-term conditions who had experienced cybervictimization. Table 1 summarizes the codes given to the interviewees and the main description of their experiences. The long-term conditions and impairments are listed in the table using the participants’ own words. From these interviews, 6 overarching themes emerged.

**Table 1:** The characteristics, demographic information, health conditions, and the cybervictimization experience of the interviewees.

Interviewee	Age (years)	Gender	Ethnicity	Long-term condition	Main cybervictimization experience
B1	27	Female	White	Diabetes type 1	Cyberstalking by an ex-partner
B2	45	Female	White	Asthma, COPD^a^, eczema, and IBS^b^	Cyberstalking and cyber harassment by a stranger
B3	36	Female	White	Congenital bone disease, asthma, eczema, gynecological problems, sinus problems, anxiety, and migraine headache	Multiple incidents of cyber harassment and web-based disability hate campaigns by strangers
B4	60	Female	White	ME^c^, fibromyalgia, and spinal injury	Cyber harassment in an online support group and web-based disability hate
B5	29	Female	Latino	Epilepsy, depression, and PTSD^d^	Cyberstalking, cyberbullying, and disability discrimination by a work manager
B6	52	Female	White	Adrenal fatigue and hypothyroidism (newly diagnosed PTSD)	Cyberstalking by ex-husband
B7	56	Female	White	Depression, essential tremor, restless leg syndrome, and diabetes type 2	Cyber harassment and cyberstalking by someone who had an affair with her husband
B8	59	Female	White	Angina, mini strokes, asthma, COPD, thyroid disease, urinary incontinence, gastric condition, and unilateral blindness	Multiple occasions of web-based disability hate, the most recent one in an online support group as part of a disability campaign
B9	40	Female	White	Thyroid disease, depression, anxiety, CFS^e^, osteoarthritis, and IBD^f^	Multiple cyber harassment incidents
B10	34	Female	White	Eczema and a mental health condition	Cyberstalking by an ex-partner
B11	51	Female	White	Asthma and bipolar disorder	Cyberstalking by a “man who got involved with”
B12	48	Male	African	Depression and another unspecified condition	Multiple cyber harassment experiences and potential cyberstalking following an incident at work
B13	53	Female	White British Anglo-Indian	Psoriasis and psoriatic arthritis	Cyberbullying by the work manager

^a^COPD: chronic obstructive pulmonary disease.

^b^IBS: irritable bowel syndrome.

^c^ME: myalgic encephalomyelitis.

^d^PTSD: posttraumatic stress disorder.

^e^CFS: chronic fatigue syndrome.

^f^IBD: inflammatory bowel disease.

### Theme 1: Biomedical Events

**Figure 2:**
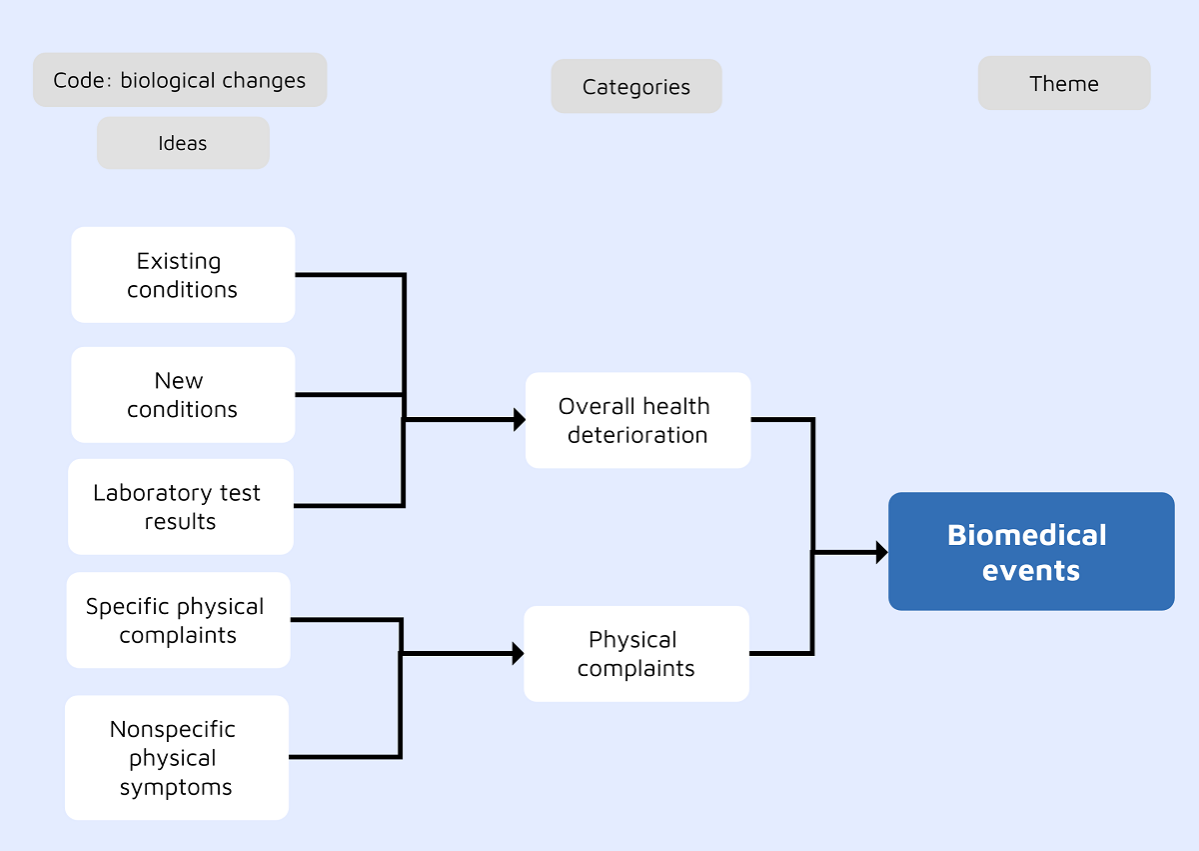
The development of the biomedical events theme.

This theme centered on the physical health consequences experienced by the participants following cybervictimization. Most participants were concerned about their chronic conditions; however, the acknowledgment of the physical impact often happened during relatively long cybervictimization campaigns. The most common category was overall health deterioration. As illustrated in Figure 2, this was perceived in relation to existing health conditions, developing new conditions, and changes in laboratory test results.

The conditions most prevalent in this sample were diabetes, asthma and chronic obstructive pulmonary disease, neurological conditions, thyroid diseases, and musculoskeletal disorders. Some participants developed new health conditions, such as migraines. The deterioration of laboratory test results was observed among patients with diabetes and thyroid disease. The following are examples of the deterioration in health:

I don’t have the full blown ones often, what I have because of my visual cortex effect. I see flashing dots all the time. That’s the type of seizures I have but sometimes very stressful situations can trigger the full-blown seizures and I’ve had those in July 2015. I had nearly thirty seizures in one week, I was sent to hospital and that was stress related so at the moment of stalking and everything I have to really be careful of this stress and that’s how it goes to full blown seizures.

Participant B5

Chronic pain of fibromyalgia and arthritis also got worse due to bullying and stalking.

Participant B9

My health suffered hugely. I was prescribed anti-depressants, but they gave me horrible nightmares on top of the ones I was already having. My weight began to increase alarmingly, despite not eating much. Lack of sleep and constant hypervigilance made me exhausted. I started getting pains in my joints and large muscles—this was my hypothyroidism and adrenal fatigue returning. I began to suffer debilitating migraines and was taking maximum doses of Sumatriptan to manage these mercifully, this medication was effective. My stalker knew full well that he would make my hypothyroid condition worse...he did the same to a previous partner when she had breast cancer. I consider his actions attempted murder. I am still trying to recover my physical (thyroid and adrenal) and mental (PTSD) health.

Participant B6

The *physical health complaints* included nonspecific health complaints such as palpitations, exhaustion, loss of consciousness, appetite and weight changes, or vomiting. In addition, specific health complaints refer to the symptoms relevant to the diagnosed health conditions. In a negative instance, a participant with a congenital bone disease did not perceive a link between being cybervictimized and their physical health.

### Theme 2: Impact on Mental Health

The participants experienced severe psychological and mental health impacts imposed by cybervictimization. In total, 2 subthemes were identified: psychological and psychiatric effects and helplessness.

In the *psychological and psychiatric effects* subtheme, the impact was observed in most participants through the sharing of a range of emotions; compulsions; exacerbation of existing mental health conditions; and the development of new ones, mainly PTSD, depression, and anxiety. For example, a participant shared the following:

Due to all the abuse and various other stressors, I had a psychotic breakdown in 2009. It was only then I sought treatment and was diagnosed...I believe I’m permanently psychologically damaged.

Participant B11

The *helplessness* subtheme reflected how the victims were overwhelmed by the experience of cybervictimization, felt that it was endless, and were struggling to deal with it:

It’s like you’ve got your hands tight you can’t fight back, there is nothing I can do at all. There is nothing I can do (crying) and she can do all this and. And there is no coming back.

Participant B7

Some participants perceived helplessness as a survival issue in which either the harasser or the victim can stay or the harasser is intentionally intimidating the victim to commit suicide. Alternatively, some participants found their own ways to fight back, for example, by adopting the social model of disability, campaigning, or approaching human rights organizations.

### Theme 3: Multifaceted Disruption

**Figure 3:**
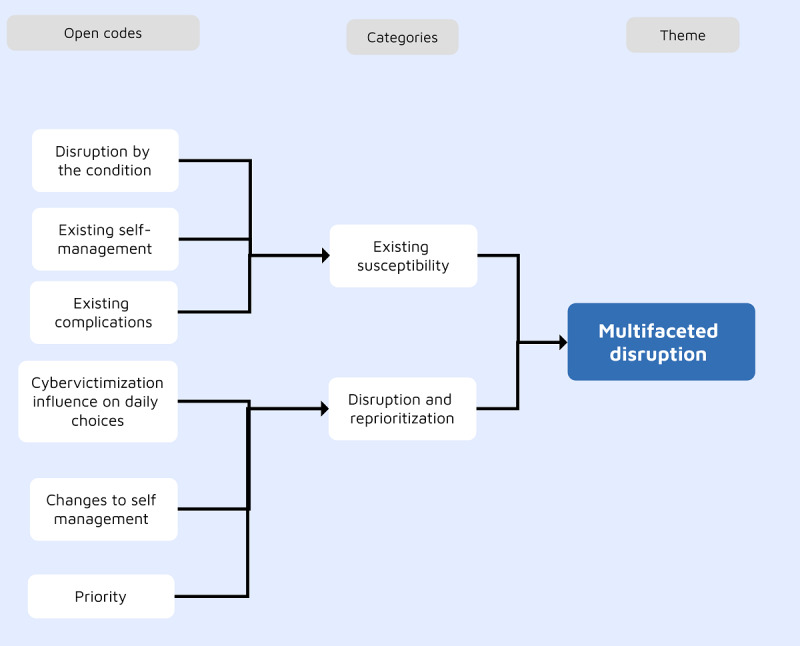
The development of the multifaceted disruption theme.

All participants perceived changes in their self-management plans of health conditions; in most cases, there were preexisting disruptive influencers on self-management, which were further disrupted by the cybervictimization experience. In total, 2 subthemes were identified: existing vulnerability and disruption and reprioritization. Figure 3 illustrates the development of this theme.

The *existing vulnerability* subtheme focused on participants’ descriptions of the continuous effort to accept the diagnosis, manage complications, and cope with them daily. Some participants incorporated their conditions by creating new web-based identities to cope. However, being entirely web-based brought about risks, too.

The *disruption and reprioritization* subtheme represents the point at which cybervictimization was introduced as a disruptive event to health. Some participants described undesirable adjustments such as changes in medications, loss of follow-up, or nonplanned lifestyle modifications. In the reprioritization process, self-management went back into the victims’ priority list, whereas cybervictimization became their primary focus. Most participants described indifference about managing their chronic conditions. The following are examples of such responses:

When it’s happening, when I’m receiving these things and when I’m stressed about it my diabetes become, goes back to my mind, like it’s not a priority so I won’t think about blood testing or I might not feel hungry, so when I have a meal I don’t do a blood test so the impact of stress on it makes me sort of forget to do that sort of side things.

Participant B1

I couldn’t manage I gave up trying and it got worse. After I gave up and took too many pain killers and didn’t have a plan for about eighteen months.

Participant B13

The disruption caused by cybervictimization also included other indirect dimensions such as unemployment, homelessness, and the closing down of electronic communication channels. A participant attempted to leave the country, but the targeting continued:

...I found work outside the UK but the mobbing continued and I had to return to the UK when my contract in Singapore finished and I couldn’t find another. On my return I entered repetitive depressive cycles due to this unemployment.

Participant B12

### Theme 4: The Impact of Complexity

This theme highlights the perceived uniqueness of individual cybervictimization cases and the survivors’ attempts to obtain formal support. In total, 2 subthemes emerged: complex situation and struggle for support.

In the *complex situation* subtheme, the process of cybervictimization was described as an ongoing cycle with on-and-off patterns and threats. The process of cybervictimization was described using different phrases by the victims, but they shared similarities in patterns. The details of each case included describing the entire experience; the harasser’s portrayal, motives, attitude, and method of contact; and the distressing content. Most of the victims could not explain the experiences they went through. The content of the messages or calls included insults, threats, sexual references, or photographs. Consequently, the pattern of contact, content, and perceived motivations left the victims in a state of anticipation and facilitated stress-induced impact. Some harassers threatened to kill themselves, whereas others threatened to hurt the victims or encouraged them to harm themselves. The following quotation is an example of such an experience:

Whilst I was struggling to survive on very low income, in inadequate housing and with an increasingly complex mental state. I felt abandoned to my fate, and that my stalker was going to achieve his aim—for me to kill myself.

Participant B6

The perceived complexity emerged from participants’ viewpoints when they felt that their situations were different from others’ because of the specifics of each case. Hence, despite being different, the victims shared the belief that their cases were unique. One aspect was related to work and organizational reputation, such as working in sensitive organizations with political involvement. Another aspect was when the suspected harassers were coworkers in well-known organizations. Some participants worked in administration in online support groups; hence, when they faced harassment, they were responsible for taking action as administrators, which triggered further harassment. Other participants thought that their cases were unique because of their history of complex home or health experiences. The following are examples of such perceived complexity:

I am constantly crying. It’s hard to get the police to take me seriously that it is still happening and to make matters worse it is to keep me silent and ruin my life as I was a victim of child sexual exploitation as a teenager, and if they keep this up, they still have power and control over me.

Participant B10

He uses the fact I can be manic occasionally, depressed a lot, anxious a lot and in particular prone to psychosis (with delusions) as a tool to help keep him from being prosecuted.

Participant B11

Some complexity aspects were related to diversity elements and being from a minority group or groups with “less power,” including ethnicity, gender, and sexual diversity. In such cases, the participants perceived that targeting was complicated by additional elements of diversity to being a woman, having a chronic condition, or being disabled.

Most victims shared that they initially underestimated the situation; they thought that it was not serious or it would end soon. However, the situation in all cases worsened, leading to safety concerns. The participants thought that the experience might not be harmful and would pass, whereas some attempted reasoning with the harassers:

I thought in a couple of weeks it will go down it will be okay but then as this is going on I’m thinking actually it is not going any better.

Participant B1

Subsequently, the victims realized that they were in danger, and most of them were concerned about facing the harasser in person and being assaulted in their homes, especially when the harassers could access the victims’ addresses using the internet or inferring them from social media or workplace information. The sense of danger affected victims’ well-being, exacerbating fear and anticipation.

The *struggle for support* subtheme reflects how approaching instrumental support channels triggered further distress rather than resolving the situation. The victims perceived hostility, ignorance, humiliation, not being taken seriously, and a lack of training. The following is an example of the impact of contacting the police:

I am constantly crying. It’s hard to get the police to take me seriously that it is still happening and to make matters worse it is to keep me silent and ruin my life.

Participant B10

Most participants did not communicate the victimization to their general practitioners (GPs), mainly because of embarrassment or because the victims did not perceive it as a health issue. Few participants thought that the GP was helpful by referring them to victims or web-based support. However, most victims thought that, despite experiencing health consequences, GPs could not help them. Their reasons were either perceiving noninterest from the GPs or insufficient training or resources:

I go to a GP, who, they are really sympathetic and everything but they don’t have the capability in order to know what to do really and they haven’t got the time to sit and talk so it’s a matter of going back to repeat prescriptions...so it’s a tick list really, so I think for people like myself, and people who needs support for things like this, It’s not there, is no sort of mental health support, there is no funding or anything so.

Participant B7

Some participants blamed their GPs for referring them to web-based support where the victimization occurred, whereas others considered that a referral to web-based support was an approach to keep patients away from GPs. Some victims started seeing therapists and counselors; however, some participants thought that counseling made things worse.

### Theme 5: Social Network Involvement

**Figure 4:**
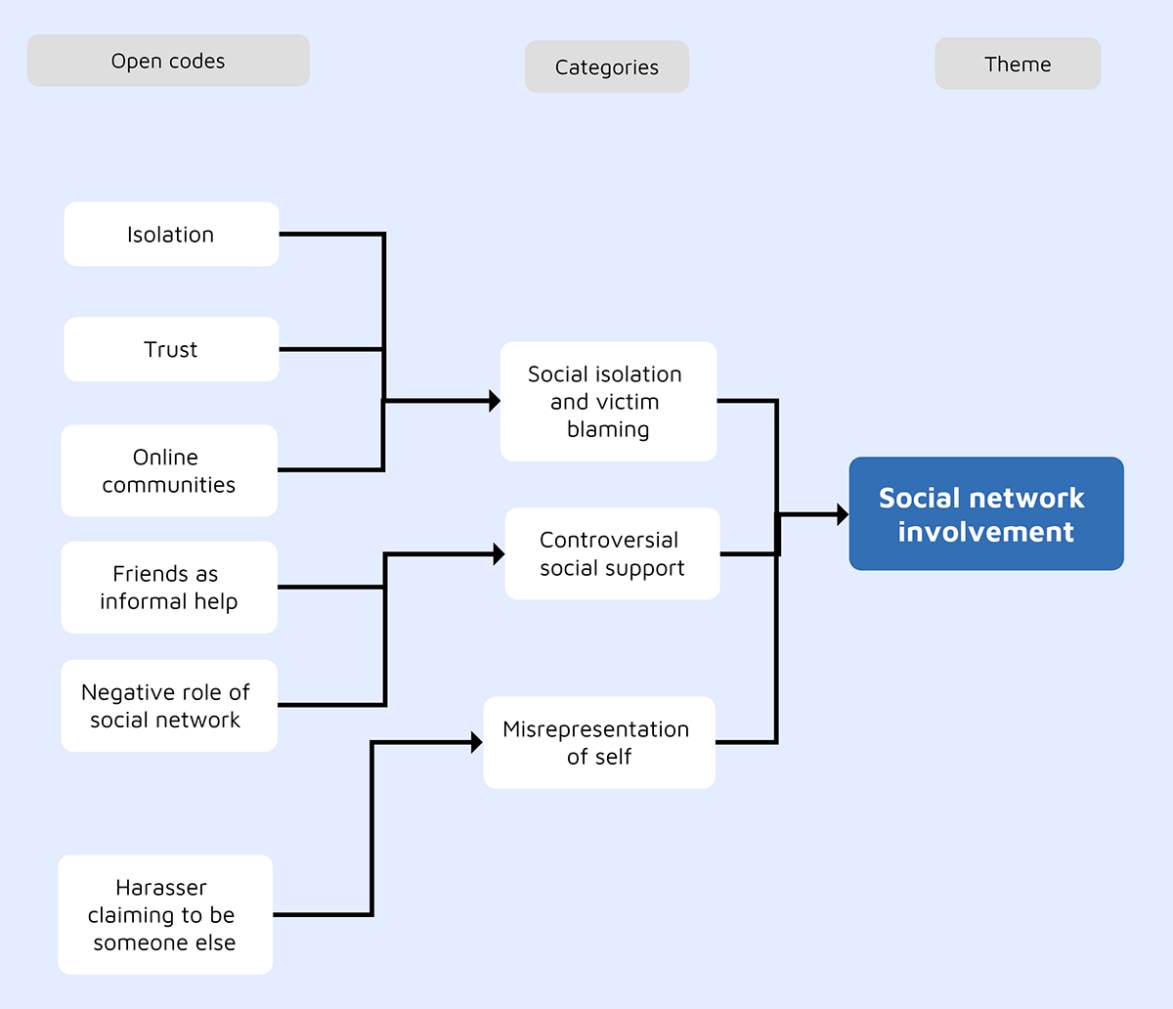
The development of the social network involvement theme.

The social network under this theme includes family, friends, web-based communities, and other social interactions. In total, 3 subthemes were identified, as illustrated in Figure 4.

The *social isolation and* victim *blaming* subtheme emerged from responses in which the cybervictimization experiences became known to family and friends. Most victims experienced social isolation and described people disappearing from their lives. Some victims were faced with statements underestimating the harassment in an web-based context, such as it being funny or unrealistic. Many victims felt betrayed and lost trust in people and society. For example, participants said the following:

I don’t have any support. I feel, to be honest people shy away from it, they they, it’s like victim blaming, do you know what I mean, you kind of brought it on yourself or they don’t know how to deal with it so they don’t deal with it they just cut you off. [Participant B2]

The people on twitter could see what was happening, and you know, none of my circle friends said you know I’m coming over and bringing take away.

Participant B3

The *controversial social support* subtheme represents the variable roles played by the social network, such as providing informal support, being secondary targets, or contacting the harasser. However, this was not helpful and, in some cases, underestimated the risk. Some participants approached web-based groups to obtain health support; although some groups were supportive, harassment was perceived from other group members:

I felt betrayed, as I had tried to be supportive to them in their journeys towards their diagnoses. Eventually I left the group...I did have sleepless, tearful nights, wondering if these people were right—maybe I am lazy, maybe I do have thyroid problems.

Participant B4

In the *misrepresentation of self* subtheme, the harassers used deception to enter the victim’s social network, such as claiming to be health care professionals to obtain sensitive information, for example, therapists or psychologists. Some harassers pretended to have the same medical condition to encourage victims to share further information. Online health support groups were also misused by salesmen and advocates for alternative treatments.

### Theme 6: Disability Discrimination

**Figure 5:**
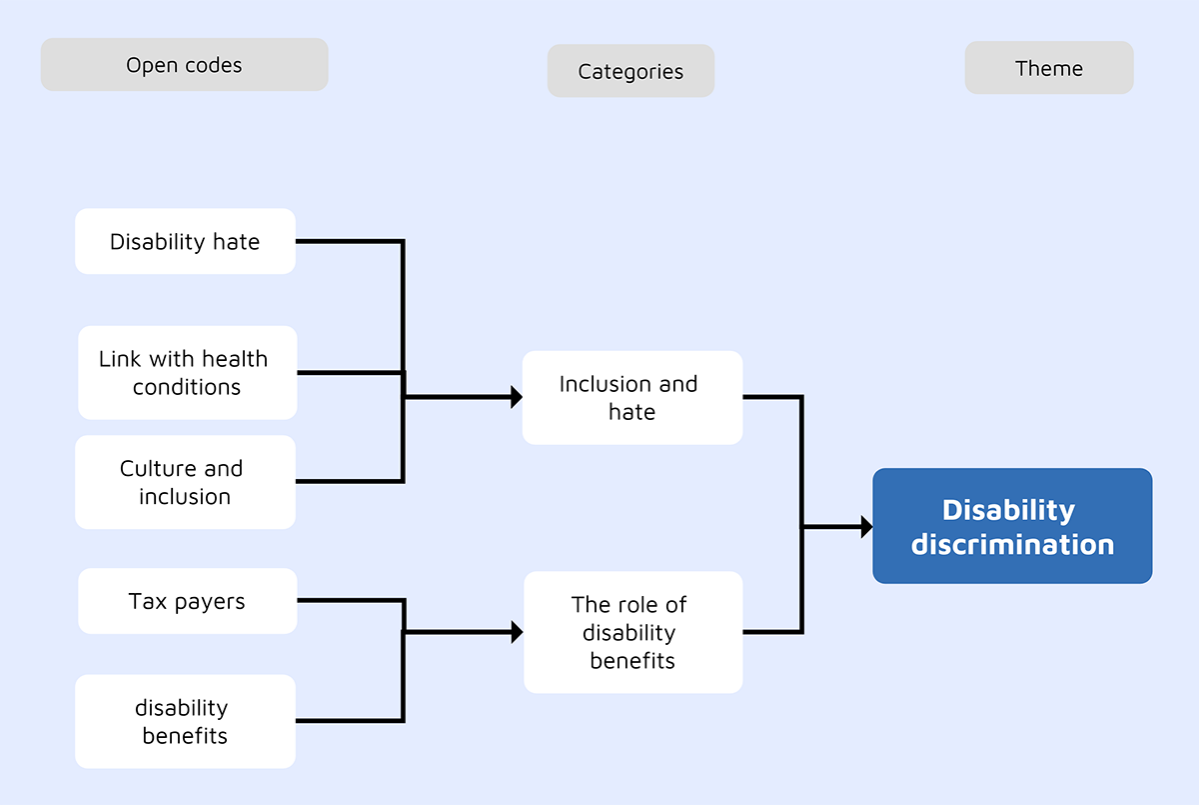
The development of the disability discrimination theme.

This theme emerged from the responses of participants with disabilities living with visible and invisible impairments. This theme reflected participants’ experiences with disability discrimination, in which they faced hate campaigns by individuals or groups. In total, 2 subthemes were identified: inclusion and hate and the role of disability benefits, as shown in Figure 5.

In the *inclusion and hate* subtheme, participants were faced with abusive web-based behavior that was perceived to be underlined by disability prejudice. Disability hate and targeting were experienced, ranging from offensive words to organized campaigns. For example, a participant shared the following:

You know when you know wake up in the morning and umm your twitter’s notifications has got hundreds of messages from at least two dozens different people because umm one person who has got 7000 twitter followers has decided hey let’s abuse this disabled person.

Participant B3

The participants were subjected to offensive language, usually by multiple harassers, and in some cases, participants were told to kill themselves or that they should be beaten or left to starve to death. Most participants who experienced this referred to it as a hate incident or crime.

The medical condition was used when the harassers tried to find the victims’ medical information over the web and then used it to abuse them. Another form of cybervictimization was that, when the harassers did not find information on physical illnesses over the web, they used the information they found over the web about mental illnesses to claim that these persons with disabilities were not genuinely disabled. Some participants experienced these comments when they used web-based shopping services, and this abuse affected how they dealt with their physical impairments afterward.

The medical legitimization of the impairment to the public was one of the prominent findings. Some participants experienced cybervictimization because of the relative invisibility of their conditions (ie, they were harassed because of not looking physically disabled), so they had to legitimize their conditions to “others”:

What is most frustrating is that chronic illness is never ever mentioned, it’s never mentioned at most of people on have a chronic illness, it’s not a physical disability that puts them on a wheelchair, we need to talk about that and also need to talk about the fact that most of the disabilities are hidden disabilities we need to have conversation on that.

Participant B8

To overcome this, when the participants had multiple conditions, the apparent physical impairments were used to legitimize the disability to the public to avoid harassment. An approach to avoid harassment was through creating web-based identities in which the disability was incorporated, as highlighted by one of the participants:

I have cultivated a scatter brained persona and nickname...and cover up my problems with humour. For that reason, I do prefer to interact online, as it is easier to sit and wait for the right word to come to mind, or rewrite a sentence without it.

Participant B4

Participants with disabilities who experienced cybervictimization perceived that such hate incidents and the medical stereotyping of disability were underlined by cultural discrimination issues and that the surrounding society is unwelcoming to chronic illness and disability.

Such discrimination left the victims with a sense of exclusion; the surrounding culture failed to include them, and this was also extended to the web-based context. Twitter users with disabilities were labeled as lazy and reliant on others. In addition, they felt excluded because other users in their network were passive toward this harassment. The policy on Twitter at the time of harassment was not protective, either.

The *role of disability benefits* subtheme emerged from participants’ experiences of abuse that were linked to financial causes, mainly participants relying on disability benefits. Some victims perceived that their harassers’ motives were related to tax paying as they believed that people with disabilities were a waste of money. Hence, harassers’ comments included discouraging people from web-based shopping as it is more expensive or included abusive threats that people with disabilities should be beaten to discourage them from claiming benefits. Accordingly, the participants who received disability benefits were frequently harassed over the web.

Some working people with disabilities who were cybervictimized and experienced health complications had to give up their jobs and apply for benefits. However, these were facing support channels that were not adequately trained to understand the impact of cybervictimization.

Despite this, even in cases in which disability benefits were granted, most survivors faced difficult circumstances when they experienced cybervictimization. This left the participants with difficult living situations that affected their mental health, physical health, and families:

I have gone from a £350-£400/day consultant to living on benefits. One of my ex-partners aborted our unborn child to get away from me when it started to become clear supporting her would be an issue.

Participant B12

I was homeless for a while because the council wanted to put me into a flat which, even with Housing Benefit, I couldn’t afford.

Participant B6

The role of disability benefits was underlined by tax paying and the political context. The participants blamed the media and political context for promoting such hostile thoughts that mediated the harassment.

Subsequently, another form of cybervictimization was malicious calls to the disability benefit hotline. In such cases, the harassers contacted the hotline to claim that the person was pretending to be disabled. This affected the survivors, as their support was suspended to prove their innocence. This period of suspension caused much distress to the victims, with another level of impact on their health.

## Discussion

### Principal Findings

The findings of this study demonstrated the multidimensional consequences of cybervictimization and its direct and indirect influence on the self-management of chronic conditions. A total of 6 overarching themes emerged from 13 interviews. These included biomedical impacts, such as deterioration of health, complications of existing conditions, and the development of new ones. The effect on mental health was apparent, including the emergence of new mental health conditions and a feeling of helplessness. All interviewees perceived changes in their self-management plans, manifested through disrupting their ability to cope with their conditions and the need to change their priorities. The participants also experienced an impact from the perceived uniqueness and complexity of the situation in addition to the struggle to obtain support. The consequences also included social network involvement, such as experiencing social isolation, victim blaming, and deception. Disability discrimination in cyberspace emerged as a major theme. It was demonstrated through hateful language, accusations of being frauds, cyber hate campaigns to discourage people who are disabled from using taxpayers’ money, and overall a failure of inclusion in the political and media context.

### Comparison With Prior Work

The findings for each theme demonstrated different yet interconnected levels of cybervictimization impact. Theme 1 focused on the deterioration of physical health, possibly a collective result of the disruption precipitated by all other themes. The physical repercussions confirm the findings from quantitative research with children and young adults, such as subjective health complaints documented in previous studies [21], which are consistent with the symptoms described in the *physical complaints* subtheme. These symptoms could be further explained by the psychosomatic experiences presented in theme 2 and their effect on mental health. These complications signify the importance of raising awareness of the impact of cybervictimization on health among the public and support channels.

In theme 1, the realization of the physical ramifications was relatively late, possibly because of prioritizing the cybervictimization experience, as seen in the reprioritization aspect discussed under theme 3 and the multifaceted disruption. Such a late realization of the deterioration of health might also explain the statistically significant relationship between the perceived impact of cybervictimization and the duration of harassment of a year or more, which is a finding from the quantitative phase of this study [30]. Furthermore, the overall health deterioration, specifically the complications of specific conditions and the development of new conditions, is an addition to the literature. The specific complications manifested in some patients with diabetes, asthma and COPD, neurological conditions, thyroid diseases, and musculoskeletal disorders. The participants reported the consequences but were not clinically checked as this study followed a phenomenologically informed approach in which they were viewed as experts in their own experiences [50]. This theme addresses the conditions collectively; however, it is recognized that each condition and each impairment is different, and the comorbidities can result in unique experiences that require exploration to understand the effects and provide tailored support.

Theme 2 represented the impact on mental health and its complications. This affected the self-management of health as it undermined resilience and coping, both of which are essential for managing long-term conditions [8]. The survivors reported PTSD, which was documented in the literature in apparently healthy individuals who experienced cyber abuse [70]. The repercussions on mental health confirm cybervictimization as a traumatic event. Depression and anxiety were commonly of concern, consistent with the documented impact of cybervictimization on people with chronic conditions and disabilities. This has mostly been documented in young people in cross-sectional studies [19]. Hence, this study confirmed the disruptive impact of cybervictimization experiences on mental health from previous studies and extended it to adults with long-term conditions. The resultant symptoms and behaviors secondary to distress subsequently affect adherence to self-management, as noted in theme 3.

The participants experienced helplessness in which cybervictimization became a survival issue, and in some cases, this inspired the determination to fight back. Such an impact is consistent with the existing literature, in which self-harm is documented with cybervictimization experiences [25]. This finding, combined with the struggle to obtain support (theme 4), is of concern as the participants might further deteriorate. In a study with young people, suicide was seen as an escape route from the cybervictimization experience [42]. Consequently, improving formal support for victims is crucial. On the positive side, the realization of rights and the will to fight are a point of strength that health promotion activities and victim support organizations can build on to improve other victims’ experiences, for example, through peer support [71] as a form of social support. This also builds on the literature that shows social support as an important factor for positive outcomes [21,42,72], which aligns with how crucial social support is for the self-management of health conditions [73,74]. Another example of fighting back is adopting the social model of disability, in which the disability results from society’s prejudice [75]. This is also a point of strength to adopt in raising awareness and counteracting the disturbing discrimination seen in theme 6*.*

The multifaceted disruption in theme 3 created a situation susceptible to cybervictimization. The self-management of the long-term condition was not at a stable stage of coping, which could have predisposed the physical and mental impact on themes 1, 2, and 6. The participants described undesirable adjustments, such as changes in medications, loss of follow-up, or nonplanned lifestyle modifications, and these findings are consistent with the quantitative results of this study [30].

Furthermore, loss of communication, homelessness, and unemployment were consistent with previous studies with apparently healthy adults [28,48]. The support channels in theme 4 caused more consequences for self-management as the responses from the support channels, including the police and GPs, were not proportional to the impact, which exacerbated the consequences and undermined trust in formal support. Lack of training and support is a consistent issue in the literature [16,76]. This theme might contribute to underreporting, another challenge faced in cyber hate and disability cases [31]. These variations necessitate health promotion, well-trained staff, and an integrated approach to cyber abuse [16,77].

Part of the complexity in theme 4 could be linked to the sample demographics, for example, the potential role of the intersectionality of gender, race, or sexual orientation with disability in cybervictimization experiences. Most of the respondents in this study were White women. There were 15% (2/13) of participants from ethnic minority groups, one of whom was a man. The sample demographics may reflect the influence of sampling in the first stage of the study [30]. It can also be a factor to examine in future research as the relationship between gender and cybervictimization is inconsistent in the literature. Among young people, cybervictimization was associated with boys [78], and in other cases, it was associated with girls [79,80]. Notably, most studies focusing on victimizing people with disabilities focused on boys [24,25,29], and some studies showed increased cybervictimization toward girls [81]. The psychological effects have also been more documented among girls [26]. Further research is needed to examine whether this study reflects attitudes toward participation a greater impact of cybervictimization among women or whether cultural factors have influenced the results, for example, if men are seen as masculine figures who should not disclose similar experiences.

The involvement of the victims’ social network was represented in theme 5, and it is a commonly documented issue in cybervictimization cases [16,28,76]. In this study, cybervictimization influenced the self-management of chronic conditions in various ways. The consequence of this is the depletion of social support, which is necessary to support those who experience cybervictimization [21], and it is also an important aspect of self-management [8]. Social support was lost when the harassment was over the web or when web-based groups were not supportive. This is important when considering providing a safer web-based environment for health support. The harasser’s involvement was also through claiming to be someone else; such deception is documented against people with disabilities [16]. Harassers posing as health care professionals, such as mental health specialists and GPs, have potentially affected self-management by undermining follow-up and through loss of trust.

Disability prejudice and the determined targeting to impose harm on persons with disabilities was a troubling finding. Such hate incidents are increasingly documented, including the cyberbullying of young people [24] or adults [82] who are disabled. As such, this study is consistent with previous findings, which are alarming and require urgent multidisciplinary action. This issue is being increasingly recognized in the wider context of the United Kingdom and by the police. However, the actions taken might not be proportional to the potential harm. An example of protective actions was the recommendations for current legislation to protect people who are disabled in the United Kingdom [32] and the ongoing changes to policy [83,84]. In addition, public health emergencies such as the COVID-19 pandemic have resulted in an increase in both web-based presence and disability discrimination [85]. Nevertheless, further work is needed to tackle the wider discriminatory scene and how public health emergencies might further influence the web-based experiences of people with disabilities.

From a biographical disruption perspective [35], cybervictimization was experienced as a traumatic, disruptive event that added to the complexity of managing a long-term condition and adversely influenced the well-being of the participants. The impact on *thinking of taken for granted* could be seen in the reprioritization process in theme 3. It is also relevant to theme 1 in how the participants initially underestimated the situation and then realized the overall health deterioration. In theme 4, the participants could not explain the cybervictimization experience, and the experience left them helpless in theme 2. This could be relevant to the disruption to the *explanatory system* in biographical disruption. Theme 2 focused on the impact on mental health, and theme 5 highlighted the social isolation and victim blaming from the social network. Both interfere with *moving social and psychological resources* in the biographical disruption concept.

### Strengths and Limitations

This study has contributed to the body of literature by focusing on the cybervictimization experienced by adults with long-term conditions and using qualitative in-depth interviews. Most of the participants in this study (12/13, 92%) were female from White ethnic backgrounds, with some representation of other groups. The researchers recognize the potential impact of the recruitment process in this research [51] and the role of inviting the participants at the end of the quantitative stage [30] to the sample in this study, which could have led to the current sample demographics. Nonetheless, cultural differences can also explain this, as can the intersectionality of cyber hate issues or the loss of trust in formal support. This area requires further exploration.

The health conditions were reported within the themes. Long-term conditions and impairments are not a homogeneous group. This research, along with the quantitative phase, represents a first step in exploring this area and identifies potential conditions that require further research or specific support in practice.

Some participants were still experiencing cybervictimization during the interview process. This might have exacerbated the extent of the reported effects; however, the researchers see this as a strength as it represents what the situation “feels like” and how the impact on well-being is experienced. The results show that cybervictimization is a disruptive event that affects self-management and could be explained from the perspective of biographical disruption [35]. However, in participants with congenital impairments or very early diagnoses, the effects in theme 3 might not be fully understood as they had a longer time for coping and potentially for self-management [37]. Nevertheless, they still experienced physical and mental health consequences.

### Conclusions and Future Directions

This study revealed the disruptive impact of cybervictimization on people with chronic conditions and disabilities. These effects were biomedical, mental, and multilevel, complicated by social and societal involvement. This study established this research area in the United Kingdom to share peoples’ voices, raise awareness, and build proper support for people with long-term conditions. This study is a step forward in a long journey to examine this phenomenon and initiate change. It signifies the need for multidisciplinary research and multiagency efforts to address this issue. The findings of this study were used to increase awareness in the United Kingdom, and because of the similarities in cybervictimization experiences documented in other countries [14,17,27], these findings could inform the support provided to survivors worldwide. The impact on health was consistent overall, and further research on the consequences for specific conditions is recommended. This work can inform practice and training for health care professionals to provide meaningful support. Future interventions can also build on positive points for support, such as fighting back and social support. Raising awareness among the public could help them understand how *real* cybervictimization experiences feel, the impact of victim blaming, and the role of contextual factors in disability discrimination.

## Abbreviations

GP: general practitioner
PTSD: posttraumatic stress disorder

## Data Availability

The data set was not placed in an open repository owing to the sensitivity of the topic.

## References

[1] Alwan A (2011). Global status report on noncommunicable diseases 2010. World Health Organization.

[2] (2020). People with long-term health conditions, UK: January to December 2019. Office for National Statistics.

[3] (2019). State of health in the EU: United Kingdom country health profile 2019. European Observatory on Health Systems and Policies.

[4] Gulley SP, Rasch EK, Chan L (2011). The complex web of health: relationships among chronic conditions, disability, and health services. Public Health Rep.

[5] (2012). Long Term Conditions Compendium of Information: Third Edition. Department of Health.

[6] Kirk-Wade E (2022). UK disability statistics: prevalence and life experiences. The House of Commons Library.

[7] Equality Act 2010: guidance on matters to be taken into account in determining questions relating to the definition of disability. Office for Disability Issues, HM Government.

[8] Greenhalgh T (2009). Chronic illness: beyond the expert patient. BMJ.

[9] Kralik D, Koch T, Price K, Howard N (2004). Chronic illness self-management: taking action to create order. J Clin Nurs.

[10] Newman S, Steed L, Mulligan K (2004). Self-management interventions for chronic illness. Lancet.

[11] Hinder S, Greenhalgh T (2012). "This does my head in". Ethnographic study of self-management by people with diabetes. BMC Health Serv Res.

[12] Sattoe JN, Bal MI, Roelofs PD, Bal R, Miedema HS, van Staa A (2015). Self-management interventions for young people with chronic conditions: a systematic overview. Patient Educ Couns.

[13] Franklin M, Lewis S, Willis K, Bourke-Taylor H, Smith L (2018). Patients' and healthcare professionals' perceptions of self-management support interactions: systematic review and qualitative synthesis. Chronic Illn.

[14] Fridh M, Köhler M, Modén B, Lindström M, Rosvall M (2018). Subjective health complaints and exposure to peer victimization among disabled and non-disabled adolescents: a population-based study in Sweden. Scand J Public Health.

[15] Sentenac M, Gavin A, Gabhainn SN, Molcho M, Due P, Ravens-Sieberer U, Matos MG, Malkowska-Szkutnik A, Gobina I, Vollebergh W, Arnaud C, Godeau E (2013). Peer victimization and subjective health among students reporting disability or chronic illness in 11 Western countries. Eur J Public Health.

[16] Alhaboby ZA, al-Khateeb HM, Barnes J, Short E (2016). ‘The language is disgusting and they refer to my disability’: the cyberharassment of disabled people. Disabil Soc.

[17] Kowalski RM, Giumetti GW, Schroeder AN, Lattanner MR (2014). Bullying in the digital age: a critical review and meta-analysis of cyberbullying research among youth. Psychol Bull.

[18] Kwan I, Dickson K, Richardson M, MacDowall W, Burchett H, Stansfield C, Brunton G, Sutcliffe K, Thomas J (2020). Cyberbullying and children and young people's mental health: a systematic map of systematic reviews. Cyberpsychol Behav Soc Netw.

[19] Alhaboby ZA, Barnes J, Evans H, Short E (2019). Cyber-victimization of people with chronic conditions and disabilities: a systematic review of scope and impact. Trauma Violence Abuse.

[20] Wells M, Mitchell KJ (2013). Patterns of internet use and risk of online victimization for youth with and without disabilities. J Spec Educ.

[21] Fridh M, Lindström M, Rosvall M (2015). Subjective health complaints in adolescent victims of cyber harassment: moderation through support from parents/friends - a Swedish population-based study. BMC Public Health.

[22] Vismara M, Girone N, Conti D, Nicolini G, Dell’Osso B (2022). The current status of cyberbullying research: a short review of the literature. Curr Opin Behav Sci.

[23] Kowalski RM, Fedina C (2011). Cyber bullying in ADHD and Asperger syndrome populations. Res Autism Spectr Disord.

[24] Didden R, Scholte RH, Korzilius H, de Moor JM, Vermeulen A, O'Reilly M, Lang R, Lancioni GE (2009). Cyberbullying among students with intellectual and developmental disability in special education settings. Dev Neurorehabil.

[25] Yen CF, Chou WJ, Liu TL, Ko CH, Yang P, Hu HF (2014). Cyberbullying among male adolescents with attention-deficit/hyperactivity disorder: prevalence, correlates, and association with poor mental health status. Res Dev Disabil.

[26] Gibson-Young L, Martinasek MP, Clutter M, Forrest J (2014). Are students with asthma at increased risk for being a victim of bullying in school or cyberspace? Findings from the 2011 Florida youth risk behavior survey. J Sch Health.

[27] Annerbäck EM, Sahlqvist L, Wingren G (2014). A cross-sectional study of victimisation of bullying among schoolchildren in Sweden: background factors and self-reported health complaints. Scand J Public Health.

[28] Sheridan LP, Grant T (2007). Is cyberstalking different?. Psychol Crime Law.

[29] Sofronoff K, Dark E, Stone V (2011). Social vulnerability and bullying in children with Asperger syndrome. Autism.

[30] Alhaboby ZA, Barnes J, Evans H, Short E (2023). Cybervictimization of adults with long-term conditions: cross-sectional study. J Med Internet Res.

[31] Alhaboby ZA, Al-Khateeb HM, Barnes J, Jahankhani H, Pitchford M, Conradie L, Short E (2021). Cyber-disability hate cases in the UK: the documentation by the police and potential barriers to reporting. Proceedings of the 13th International Conference on Global Security, Safety and Sustainability.

[32] (2019). Online abuse and the experience of disabled people 2019. House of Commons.

[33] Davidson J, Livingston S, Jenkins S, Gekoski A, Choak C, Ike T, Phillips K (2019). Adult online hate, harassment and abuse: a rapid evidence assessment. UK Council for Internet Safety.

[34] (2019). Adult online hate, harassment and abuse: a rapid evidence assessment. United Kingdom Government.

[35] Bury M (1982). Chronic illness as biographical disruption. Sociol Health Illn.

[36] Giddens A (1979). Central Problems in Social Theory: Action, Structure and Contradiction in Social Analysis.

[37] Williams S (2001). Chronic illness as biographical disruption or biographical disruption as chronic illness? Reflections on a core concept. Sociol Health Illn.

[38] Larsson AT, Grassman EJ (2012). Bodily changes among people living with physical impairments and chronic illnesses: biographical disruption or normal illness?. Sociol Health Illn.

[39] Kleinman A (1988). The Illness Narratives: Suffering, Healing, And the Human Condition.

[40] Lonardi C (2007). The passing dilemma in socially invisible diseases: narratives on chronic headache. Soc Sci Med.

[41] Revenson TA, Schiaffino KM, Majerovitz SD, Gibofsky A (1991). Social support as a double-edged sword: the relation of positive and problematic support to depression among rheumatoid arthritis patients. Soc Sci Med.

[42] Dennehy R, Meaney S, Cronin M, Arensman E (2020). The psychosocial impacts of cybervictimisation and barriers to seeking social support: young people’s perspectives. Child Youth Serv Rev.

[43] Richard AA, Shea K (2011). Delineation of self-care and associated concepts. J Nurs Scholarsh.

[44] Morden A, Jinks C, Ong BN (2017). Temporally divergent significant meanings, biographical disruption and self-management for chronic joint pain. Health (London).

[45] Dreßing H, Bailer J, Anders A, Wagner H, Gallas C (2014). Cyberstalking in a large sample of social network users: prevalence, characteristics, and impact upon victims. Cyberpsychol Behav Soc Netw.

[46] Short E, Linford S, Wheatcroft JM, Maple C (2014). The impact of cyberstalking: the lived experience - a thematic analysis. Stud Health Technol Inform.

[47] Alonzo AA (2000). The experience of chronic illness and post-traumatic stress disorder: the consequences of cumulative adversity. Soc Sci Med.

[48] Maple C, Short E, Brown A, Bryden C, Salter M (2012). Cyberstalking in the UK: analysis and recommendations. Int J Distrib Syst Technol.

[49] Galeazzi GM, Bučar-Ručman A, DeFazio L, Groenen A (2009). Experiences of stalking victims and requests for help in three European countries. A survey. Eur J Crim Pol Res.

[50] Giorgi A (2009). The Descriptive Phenomenological Method in Psychology: A Modified Husserlian Approach.

[51] Alhaboby ZA, Barnes J, Evans H, Short E (2017). Challenges facing online research: Experiences from research concerning cyber-victimisation of people with disabilities. Cyberpsychology.

[52] Siddiqui N, Anneke Fitzgerald J (2014). Elaborated integration of qualitative and quantitative perspectives in mixed methods research: a profound enquiry into the nursing practice environment. Int J Mult Res Approaches.

[53] Krumholz HM, Curry LA, Bradley EH (2011). Survival after acute myocardial infarction (SAMI) study: the design and implementation of a positive deviance study. Am Heart J.

[54] De Korte-Verhoef MC, Pasman HR, Schweitzer BP, Francke AL, Onwuteaka-Philipsen BD, Deliens L (2014). General practitioners' perspectives on the avoidability of hospitalizations at the end of life: a mixed-method study. Palliat Med.

[55] Palinkas LA, Horwitz SM, Green CA, Wisdom JP, Duan N, Hoagwood K (2015). Purposeful sampling for qualitative data collection and analysis in mixed method implementation research. Adm Policy Ment Health.

[56] Britten N (1995). Qualitative interviews in medical research. BMJ.

[57] Groenewald T (2004). A phenomenological research design illustrated. Int J Qual Methods.

[58] Greenhalgh T, Campbell-Richards D, Vijayaraghavan S, Collard A, Malik F, Griffin M, Morris J, Claydon A, Macfarlane F (2011). New models of self-management education for minority ethnic groups: pilot randomized trial of a story-sharing intervention. J Health Serv Res Policy.

[59] Quarmby K (2015). Disability hate crime motivation survey-results. katharine Quarmby.

[60] Sentenac M, Arnaud C, Gavin A, Molcho M, Gabhainn SN, Godeau E (2012). Peer victimization among school-aged children with chronic conditions. Epidemiol Rev.

[61] Sentenac M, Gavin A, Arnaud C, Molcho M, Godeau E, Nic Gabhainn S (2011). Victims of bullying among students with a disability or chronic illness and their peers: a cross-national study between Ireland and France. J Adolesc Health.

[62] Reyns BW, Englebrecht CM (2014). Informal and formal help-seeking decisions of stalking victims in the United States. Crim Justice Behav.

[63] Guest G, MacQueen KM, Namey EE (2012). Applied Thematic Analysis.

[64] Fitzpatrick R, Boulton M (1996). Qualitative research in health care: I. The scope and validity of methods. J Eval Clin Pract.

[65] Phillippi J, Lauderdale J (2018). A guide to field notes for qualitative research: context and conversation. Qual Health Res.

[66] Braun V, Clarke V (2006). Using thematic analysis in psychology. Qual Res Psychol.

[67] Seale C (2012). Researching Society and Culture. 3rd edition.

[68] MacQueen KM, McLellan-Lemal E, Bartholow K, Milstein B, Guest G, MacQueen KM (2008). Team-based codebook development: structure, process, and agreement. Handbook for Team-Based Qualitative Research.

[69] Guest G, MacQueen KM, Namey EE, Guest G, MacQueen KM, Namey EE (2012). Validity and reliability (credibility and dependability) in qualitative research and data analysis. Applied Thematic Analysis.

[70] Mateu A, Pascual-Sánchez A, Martinez-Herves M, Hickey N, Nicholls D, Kramer T (2020). Cyberbullying and post-traumatic stress symptoms in UK adolescents. Arch Dis Child.

[71] Fisher EB, Earp JA, Maman S, Zolotor A (2010). Cross-cultural and international adaptation of peer support for diabetes management. Fam Pract.

[72] Hellfeldt K, López-Romero L, Andershed H (2019). Cyberbullying and psychological well-being in young adolescence: the potential protective mediation effects of social support from family, friends, and teachers. Int J Environ Res Public Health.

[73] Brouwer AM, Salamon KS, Olson KA, Fox MM, Yelich-Koth SL, Fleischman KM, Hains AA, Davies WH, Kichler JC (2012). Adolescents and type 2 diabetes mellitus: a qualitative analysis of the experience of social support. Clin Pediatr (Phila).

[74] Gallant MP (2003). The influence of social support on chronic illness self-management: a review and directions for research. Health Educ Behav.

[75] Bingham C, Clarke L, Michielsens E, Van de Meer M (2013). Towards a social model approach?: British and Dutch disability policies in the health sector compared. Pers Rev.

[76] al-Khateeb HM, Epiphaniou G, Alhaboby ZA, Barnes J, Short E (2017). Cyberstalking: investigating formal intervention and the role of corporate social responsibility. Telemat Inform.

[77] Fazio LD, Galeazzi GM Women victims of stalking and helping professions: recognition and intervention in the Italian context 2004. U.S. Department of Justice.

[78] Hong JS, Kim DH, Thornberg R, Kang JH, Morgan JT (2018). Correlates of direct and indirect forms of cyberbullying victimization involving South Korean adolescents: an ecological perspective. Comput Human Behav.

[79] Morin HK, Bradshaw CP, Kush JM (2018). Adjustment outcomes of victims of cyberbullying: the role of personal and contextual factors. J Sch Psychol.

[80] Smith PK, López-Castro L, Robinson S, Görzig A (2019). Consistency of gender differences in bullying in cross-cultural surveys. Aggress Violent Behav.

[81] Emerson E, Aitken Z, King T, Arciuli J, Llewellyn G, Kavanagh AM (2022). The association between disability and risk of exposure to peer cyber victimisation is moderated by gender: cross-sectional survey. Disabil Health J.

[82] Burch L (2018). ‘You are a parasite on the productive classes’: online disablist hate speech in austere times. Disabil Soc.

[83] Law C (2020). Harmful online communications: the criminal offences- a consultation paper. Law Commission.

[84] Law C (2020). Hate crime laws: a consultation paper. Law Commission.

[85] Shoib S, Philip S, Bista S, Saeed F, Javed S, Ori D, Bashir A, Chandradasa M (2022). Cyber victimization during the COVID-19 pandemic: a syndemic looming large. Health Sci Rep.

